# Seed size plasticity in response to embryonic lethality conferred by ectopic CYCD activation is dependent on plant architecture

**DOI:** 10.1080/15592324.2016.1192741

**Published:** 2016-06-10

**Authors:** E. Sornay, W. Dewitte, J. A. H. Murray

**Affiliations:** Cardiff School Biosciences, Cardiff University, Cardiff, Wales, UK

**Keywords:** Arabidopsis, CYCDs, compensation, cell cycle, nutrient allocation, plant development, plasticity, seed size, seed number

## Abstract

The size of seeds is the result of cell proliferation and growth in the three seed compartments: the embryo, endosperm and integuments. Targeting expression of the D-type cyclin CYCD7;1 to the central cell and early endosperm (*FWA:CYCD7;1*) triggered nuclear divisions and partial ovule abortion, reducing seed number in each silique and leading to increased seed size. A similar effect on seed size was observed with other segregating embryo lethal mutations, suggesting caution is needed in interpreting apparent seed size phenotypes. Here, we show that the positive effect of *FWA:CYCD7;1* on Arabidopsis seed size is modulated by the architecture of the mother plant. Larger seeds were produced in *FWA:CYCD7;1* lines with unmodified inflorescences, and also upon removal of side branches and axillary stems. This phenotype was absent from inflorescences with increased axillary floral stems produced by pruning of the main stem. Given this apparent confounding influence of resource allocation on transgenes effect, we conclude that plant architecture is a further important factor to consider in appraising seed phenotypes.

Plants have a sessile lifestyle and their development and growth respond to the environment.^1-6^ Within this context, plant morphogenesis is a plastic process that requires the integration of developmental and environmental cues. In higher plants, the reproductive phase leads to the formation of seeds, which are essential for plant dispersal and survival. Angiosperm seeds derive from a fertilized ovule and are comprised of three main compartments: the embryo derived from fertilization of a haploid egg cell, the endosperm derived from fertilization of the diploid central cell by haploid sperm cells and the integuments. Mitotic cell proliferation underpins the growth and development of all three components and is therefore essential for both embryonic and post-embryonic development.

The mitotic cell cycle of eukaryotes is composed of two alternating phases, during which DNA is first replicated (S phase) and then chromosomes are partitioned (mitosis, M), interrupted by two gaps G1 (between M and S) and G2 (between S and M). In eukaryotes, progression through the cell cycle requires the modulation of the activity of kinase complex composed of the cyclin-dependent kinase (CDK) and the regulatory subunit, cyclin (CYC).^7^

Cell cycle progression requires tight regulation that occurs at two main checkpoints: G1-to-S and G2-to-M. In *Arabidopsis*, CDKA1;1/CYCD kinases^8-10^ regulate the G1-to-S transition whereas G2-to-M transition requires the activity of CDKB kinases. These checkpoints are key steps to integrate developmental, environmental and nutritional cues and CYCDs have been shown to be direct or indirect integrators of mitogenic signaling triggered by phytohormones such as auxin and cytokinin and carbohydrates (e.g. sucrose and glucose) levels.^8-9^

We have used targeted expression of *CYCD7;1* using the highly specific *FWA* promoter to address the consequences of altering the normal pattern of divisions early in Arabidopsis seed development.^11^ The *FWA* promoter is active pre- and post-fertilization in the central cell of the female gametophyte and developing endosperm of the seed. Interestingly, targeted expression of *CYCD7;1* using the *FWA* promoter overcomes cell cycle arrest in the central cell of the female gametophyte before fertilization, resulting in the central cell becoming multinucleate with high frequency. Post-fertilization there is an acceleration of early endosperm and embryo development, although this slows after heart phase of embryo development, by which time the *FWA* promoter is no longer active. We also observed a high degree of lethality of embryos, leading to abortion.

Associated with these phenotypes provoked by *CYCD7;1* expression, we recently reported that *FWA:CYCD7;1* transgenic lines produce seeds with increased final size.^11^ These seeds display faster seedling establishment upon germination, which might benefit the fitness of this transgenic lines.^11^ We noted that a number of seed developmental mutants have similarly been reported to display either lethality or enlarged seeds.^12-18^ Previously only Fang and colleagues^17^ have reported both parameters and we considered whether these phenotypes might be causally related. Examining mutants with lethality but no obvious other connection to seed growth also showed increased seed size. This suggests that drawing conclusions from observations of altered seed size must be carefully considered alongside any sibling lethality observed.^12^

Hence in this study, we showed evidence for the trade-off between seed size and seed number in the siliques.^12-18^ Targeted *CYCD7;1* expression in the seed under the control of the *FWA* promoter was also tested using the *UAS/GAL4* system,^19^ and using these *FWA>>CYCD7;1* transgenic lines, the trade-off between seed size and number was further explored within the context of resource allocation. The number of seed pods was artificially decreased by removal of the branches of the floral stalk as well as the axillary shoots (referred as SUPRA, Fig. 1A) or increased by cutting back the primary stem to stimulate branching and therefore leading to the production of numerous seed pods (SUB, Fig. 1A). We found that these manipulations of plant architecture influenced seed size in untransformed WT plants, as well as modulating the effect conferred by *FWA>>CYCD7;1*. The mean seed size distribution of WT plants was not significantly different between seeds grown on NORMAL floral stalks or on SUPRA stems (i.e. when axillary stems were removed). However on SUB shoots where the main stem was removed to increase side branching, mean seed size was decreased by 4% (p = 0.76). In each case, overall mean seed size from the whole plant was determined as described.^11^ These results suggest that either mean seed size differs between the main inflorescence and side inflorescences or that it can be affected by limiting the resources available. However the former explanation would suggest that SUPRA seeds from the main stem only should be larger than from the NORMAL which includes both main stem and side shoot-derived seed, which was not the case, since seed size was not increased by axillary stem removal (SUPRA), in contrast to the effect of sibling abortion within the silique itself^12^ on seed size.

**Figure 1.**
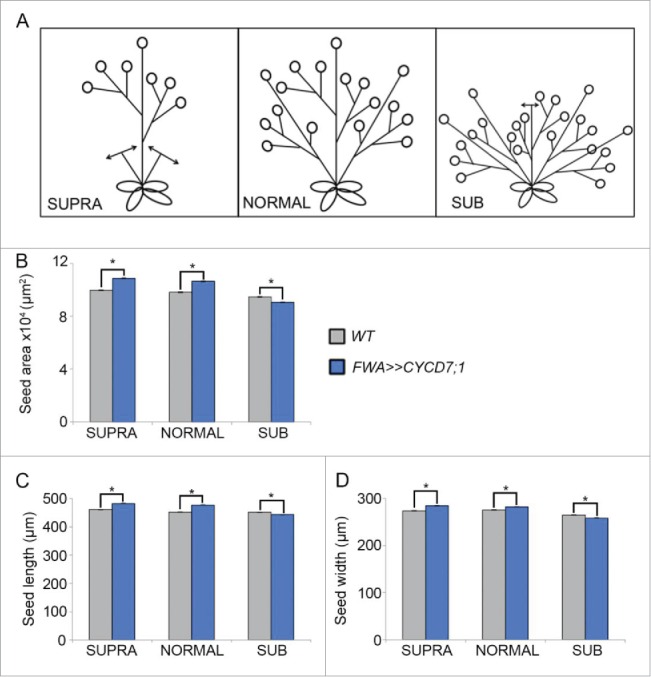
Influence of plant architecture on seed size. (A) Cartoon depicting the experimental design: in SUPRA conditions, all axillary and secondary branches were removed resulting in a reduction of the number of seed pods produced. In NORMAL conditions plant architecture was untouched. In SUB conditions, the primary stem was cut soon after the floral transition initiating the formation of additional axillary stems and increasing the number of seed pods. (B-D) Comparison of seed size parameters between the different architectures. For each type, nine plants were grown and from each individual plant seeds were harvested and analyzed seperately. For each plant, a minimum of 200 seeds was measured. Mean seed area (B), mean seed length (C) and mean seed width (D). Error bars show ± SE. (*) indicates a statistical difference in one of the of seed size parameters.

In line with our analysis of the *FWA:CYCD7;1* seeds,^11^ we found that both *FWA>>CYCD7;1* NORMAL and SUPRA plants produce larger seeds compared to WT controls (two-way ANOVA, p = 1.18 × 10^−47^; Fig. 1). In NORMAL plants, *FWA>>CYCD7;1* seeds were 8% larger than the *WT* (two-way ANOVA, p = 3.95 × 10–45) and in SUPRA conditions *FWA>>CYCD7;1* seeds showed a 9% increase in overall area. However, on the SUB plants with increased branching *FWA>>CYCD7;1* mean seed size was 4% smaller than the WT control with increased branching, indicating that the beneficial effect of targeted *CYCD7;1* expression is lost when shoot growth is stimulated. The length and width showed results similar to those observed for the overall area, suggesting that these trends are the same in both dimensions (Fig. 1). At this point we cannot exclude a possible effect of pruning on the expression of the *FWA* driven transgene, but measuring seeds from developmentally comparable shoots suggests that the effect of the transgene depends rather on the architecture of the mother's shoot.

These observations suggest that not only is seed size affected by sibling lethality but this effect can also be dependent on other aspects of growth. In this case, the manifestation of the enlarged seed phenotype conferred by *CYCD7;1* ectopic expression appears to be dependent on the availability of sufficient resources. This confounding influence of plant architecture on the effects of ectopic expression CYCD conferred phenotypes might have implications in our evaluation of phenotypes.^20-24^
